# Antiradical and Cytoprotective Properties of *Allium nutans* L. Honey Against CCL4-Induced Liver Damage in Rats

**DOI:** 10.3389/fphar.2021.687763

**Published:** 2021-09-20

**Authors:** Shynggys Sergazy, Alexander Gulyayev, Aidana Amangeldiyeva, Ayaulym Nurgozhina, Madiyar Nurgaziyev, Zarina Shulgau, Laura Chulenbayeva, Zhanagul Khassenbekova, Almagul Kushugulova, Mohamad Aljofan

**Affiliations:** ^1^ National Laboratory Astana, Nazarbayev University, Nur-Sultan, Kazakhstan; ^2^ Kazakhstan Association of Researchers of the Human Microbiome, Nur-Sultan, Kazakhstan; ^3^ National Center for Biotechnology, Nur-Sultan, Kazakhstan; ^4^ School of Medicine, Department of Biomedical Sciences, Nazarbayaev University, Nur-Sultan, Kazakhstan

**Keywords:** cytotoxicity, doxorubicin, flavonoid, polyphenol, CCl4-induced liver damage, anti-radical activities

## Abstract

The aim of this study is determine the *in vitro* and *in vivo* antiradical properties and the cytoprotective activity of *Allium nutans* L. honey extract. The antiradical properties of the extracts were investigated in rabbit alveolar macrophages and human foreskin fibroblast (hFFs) cells in the presence of doxorubicin, a cytotoxic substance using DPPH and ABTS assays. The cytoprotective activities were determined using 18 Wistar rats divided into three different groups, a negative control, and two other groups with experimentally induced hepatotoxicity by a single intraperitoneal injection of 50% carbon tetrachloride (CCl4) oil solution. A positive control group, received drinking water only and an experimental group that was treated with *Allium nutans* L. honey extracts for 7 days. *In vitro* treatment with *Allium nutans* L. honey extracts resulted in 78% reduction in radical activity in DPPH and 91.6% inhibition using the ABTS. Also, honey extracts were able to preserve 100% of cell viability in the presence of the cytotoxic, doxorubicin. Furthermore, the treatment with honey extracts resulted in a significant reduction in damage to the structure of liver tissue, as well significant reduction in the levels of ALT and AST in the experimental group compared to the control group.

## Introduction

Natural bee products are highly valued by their consumers and have been traditionally used for medical purposes as alternative therapy throughout the history of medicine. The effect of honey on humans is associated with anti-allergic, antitumor, antitoxic, antimicrobial, and antiradical activity (Alvarez-Suarez et al., 2013; Alvarez-Suarez et al., 2016). Oxidative stress plays a fundamental role in the development and spread of the state of inflammation leading to various diseases and thus, the potential protective properties of honey against diseases associated with oxidative stress have been studied quite intensively with several reported mechanisms including 1) a decrease in platelet activity, 2) prevention of LDL oxidation and 3) improvement of coronary vasodilation (Khalil and Sulaiman, 2010; Lugrin et al., 2014). Other reported therapeutic effects of honey include protection against neurotoxicity, several aging-related neurological pathologies, and gastrointestinal tract disorders have been demonstrated (Mandal and Mandal, 2011; Syarifah-Noratiqah et al., 2018).

Honey is a complex product that contains up to 400 vital components including polyphenols, which are known for their antioxidant, anti-inflammatory, and immunomodulatory properties. Honey derived phenolic compounds are classified into 10 main groups: simple phenols, phenolic acids, coumarins, isocoumarins, naphthoquinones, xanthones, stilbenes, anthraquinones, flavonoids, and lignans. However, there are inconsistencies in the therapeutic efficacy and the safety of different honey phenolic groups, which appears to depend on the region of honey origins, which was suggested to be significantly impacted by the variety of the composition of bee products, which depends on climate, geography, and bee species (Khalil and Sulaiman, 2010; Syarifah-Noratiqah et al., 2018).

There is no available data or experimental results about the properties of the original Kazakhstani bee products including the rare variety of monofloral honey such as that of the wild Allium nutans L. that grows in the Altai mountains (Eastern region of the Republic of Kazakhstan). Allium nutans L. are a rare perennial herb belonging to the genus of Allium L. and family of Amaryllidaceae that are commonly used by different cultures for their potential therapeutic values. In nature, Allium nutans L. grow on stony soils of the Altai Mountains and have been commonly used for centuries as a treatment for different diseases in the Altai folk medicine. Therefore, we hypothesized that polyphenolic extracts of the Allium nutans L. honey to likely contain important therapeutic values. Thus, the present study aims to investigate the composition and potential cytoprotective properties of the polyphenolic extract of the rare variety of monofloral honey collected from the flowering of wild Allium nutans L.

## Materials and Methods

### Ethics Approval

Animal experiments were made in agreement with “the rules of pre-clinical studies” (approved by order of the Minister of Health and Social Development Republic of Kazakhstan on May 29, 2015 № 415) and approved by the Ethical Committee of Nazarbayev University (Ethical approval № 18 from April 02, 2015). The authors confirm that the study was carried out in compliance with the ARRIVE guidelines.

### *Allium nutans* L Honey

The subject of the study was monofloral honey collected from *Allium nutans* L. (Siberian chives), which are perennial herbaceous plants endemic to the Altai Mountains region in the eastern part of Kazakhstan. As a comparison, we used a different type of honey from the same geographic region, monofloral honey, *Helianthus annuus* L. (sunflower), obtained from the area sown to sunflowers. *Helianthus annuus* L. honey is the single most widespread and standardized sample of monofloral honey in Kazakhstan. *Allium nutans* L. honey was collected in the mountains (the apiary was located during June and July during the flowering of wild slime onions at an altitude of about 1,500 m above sea level). *Helianthus annuus* L. honey was obtained by moving the apiary to the foothills in this region (300 m above sea level next to the fields of cultivated sunflower). Studied honey has passed state certification as a food product in the Republic of Kazakhstan.

### Polyphenol Extraction

The extraction of phenolic compounds from honey was performed by liquid extraction using ethyl acetate as described by Campone et al. and Pyrzynska et al. (Pyrzynska and Biesaga, 2009; Campone et al., 2014). The extract was evaporated on a rotary evaporator. The extraction procedure was performed three times. The combined extract was dissolved in 1 ml of water: ethanol (1:1) and then filtered through 0.2-micron-nylon filters (Uniprep, Whatman). The resulting solution was used for chromatographic analysis for phenolic compounds. For studies on cell culture and studies on rats, an aqueous extract of phenolic compounds from honey (without ethanol) was prepared according to the method described above. 100 ml of the extract was prepared from 50 g of honey.

### Chromatography Analysis of Phenolic Compounds

The extracted polyphenolic compounds were analyzed by a high-throughput liquid chromatography method using Agilent 1,290 Infinity chromatograph. The separation in gradient mode was performed on the column ZORBAX RRHD, SB-C18, 2.1 × 100 mm, 1.8 mcm. The mobile phase consisted of an aqueous solution of 0.1% formic acid (Solvent A) and acetonitrile containing 0.1% formic acid (Solvent B). The registration was performed by a diode array detector at 280 and 325 nm. When analyzing the honey extract, the gradient was carried out in the following sequence: the initial eluent consisted of component A. Within 5 minutes, the concentration of component B increased to 10% and remained for the next 5 minutes. From the 10th to the twenty-fifth minute, the concentration of component B increased to 35%. From the twenty-fifth minute to the forty-fifth, the concentration of component B increased to 100%. The column was then cleaned and conditioned. The flow rate is 0.3 ml/min at 30°C. For the analysis, 3 μl of the sample was used. The absorption spectrum was recorded in the range of 210–400 nm with a rate of 2 nm.

The ability of honey to neutralize free radicals *in vitro* was determined using two of the most commonly used biochemical tools that evaluate the presence of antiradical properties *in vitro* namely, 2,2-azinobis (3-ethylbenzothiazoline-6-sulfonic acid) (ABTS) and 2,2-diphenyl-1-picrylhydrazyl (DPPH) (Re et al., 1999; Truong et al., 2007) utilizing stable trolox and DPPH, respectively as the reference substances (Truong et al., 2007). For the DPPH assay, the antiradical activity was defined as the amount of antioxidant required to reduce the initial DPPH concentration by 50% (effective concentration = EC50). The ABTS test was performed according to R. Re et al. (Re et al., 1999). Briefly, ABTS assay measures the relative ability of antioxidants to scavenge the ABTS generated in aqueous phase, as compared with a Trolox and the antioxidant activity was recorded in micromoles of trolox-equivalent per ml (mmol TE/ml) (Erel, 2004).

### Cytoprotective Effects of Extracts From *Allium nutans* L. Honey

An MTT cytoprotective assay (Sigma Chemical Co., St. Louis, MO) was used to determine the *in vitro* cytotoxic effects of extracts from the *Allium nutans* L. honey using two different cell types, primary rabbit alveolar macrophages and human foreskin fibroblast cell line (hFFs) that were provided by Laboratory of Bioengineering and Regenerative Medicine, National Laboratory Astana at Nazarbayev University.

To induce cytotoxicity, cells were treated with doxorubicin at two concentrations; 1 mcg/ml and 10 mcg/ml. Simultaneously, rabbit alveolar macrophages and hFFs cells were individually treated with extract of the *Allium nutans* L. honey at 1:10 and 1:100 dilutions for 24 h. Upon the completion of treatment incubation period cells were analyzed using the MTT assay according to the manufacturer’s guidelines. Briefly, cells were treated with 3-(4,5-dimethylthiazol-2-yl)-2,5-diphenyltratrazolium bromide (MTT) (Sigma Chemical Co., St. Louis, MO) and incubated for 4 h. After incubation period, formazan crystals were dissolved by MTT solubilization solution (Sigma Chemical Co., St. Louis, MO) and the absorbance was measured at 570 nm using a 96 well-imaging reader (Cytation™ 5, Bio-Tek Instruments, Inc.). The cytoprotective index was determined using the untreated cells as negative control.

### Experimental Design: Induction of Acute Hepatitis

A total of 18 healthy adult *Wistar* rats aged ten to 12 weeks and weighing 240 ± 20 g were housed in the animal facility of the National Center for Biotechnology, Nur-Sultan, Kazakhstan. After a 10-days adaptation period, the rats were randomly divided into three groups (6 rats/cage) and housed in a room with controlled temperature and a 12-h light-dark cycle with unlimited access to standard food and drinking water ad libitum.

The hepatitis model was experimentally induced by a single intraperitoneal administration of intraperitoneal injection of 50% carbon tetrachloride (CCl4) oil solution at a dose of 0.3 ml per rat (Alhassan et al., 2009). The animals were divided into three groups; 1) experimental group; rats received a daily dose of 0.5 ml of *Allium nutans* L. honey extract via intragastric administration for 7 days prior CC14 injection (induction of hepatitis) and continued for another 14 days after (total 21 days); 2) vehicle control group; followed the same treatment period (7 days before hepatitis induction and 14 days after) and route of administration (intragastric), except the animals were given drinking water; 3) control group; no treatment or disease induction. On the 22nd day (1 day after the last administration of the honey extract), the rats were euthanized with carbon dioxide and organs were collected for further analyses. Rats (males 240 ± 20 g) were administered orally daily in a dose of 0.5 ml of honey extract, or on average 2 ml/kg. When converted to the mass of honey—1 g/kg. When choosing a dose regimen, we were guided by similar experimental studies on rats, where doses of honey from 0.5 to 1 g/kg until 5 g/kg are commonly used (Al-Yahya et al., 2013) (El Denshary et al., 2012).

### Histological Analyses

Liver samples were preserved in 10% neutral buffered formalin solution for 24 h, and then washed with 70% ethanol. Samples were then placed metal caskets, dried using 100% alcohol, and then embedded into paraffin blocks, which were sectioned and placed on glass slides to dry overnight. The slides were then stained with hematoxylin and eosin and analysed using Axioskop 40 microscope (Carl Zeiss, Germany) by two different histopathologists, which used a semiquantitative scale: normal = 0, mild = < 25%, moderate = 25–50% and severe = >50% of affected area.

### Biochemical Analyses

Fresh blood samples were obtained from vena cava for biochemical analyses.

### Measurement of Liver Injury

The extent of liver injury was determined via measuring the serum level of aspartate aminotransferase (AST) and alanine aminotransferase (ALT), which are commonly used markers of liver injury (McGill). The levels of the two markers were determined using commercially available kits AsAT-Vital kit (Vital Diagnostics SPb, Russia), and Alanine aminotransferase Activity Assay Kit (sigma-Aldrich), respectively. Another measured marker of hepatitis injury was total bilirubin, which was determined using the colorimetric Bilirubin Assay Kit (Sigma-Aldrich). In addition, total protein and glucose were determined by the commercially available kits from Sigma-Aldrich, Total Protein Kit-Micro, and High Sensitivity Glucose Assay Kit, respectively.

### Statistical Analyses

The statistical analysis of the results was performed using the Statistica 6.0 software package (TIBCO Data Science: www.tibco.com). The results are presented as “average value ±standard error of the average value”. Student’s *t*-test was used to test the difference in means.

## Results

### Comparison of Honey Polyphenol Contents

**FIGURE 1:**
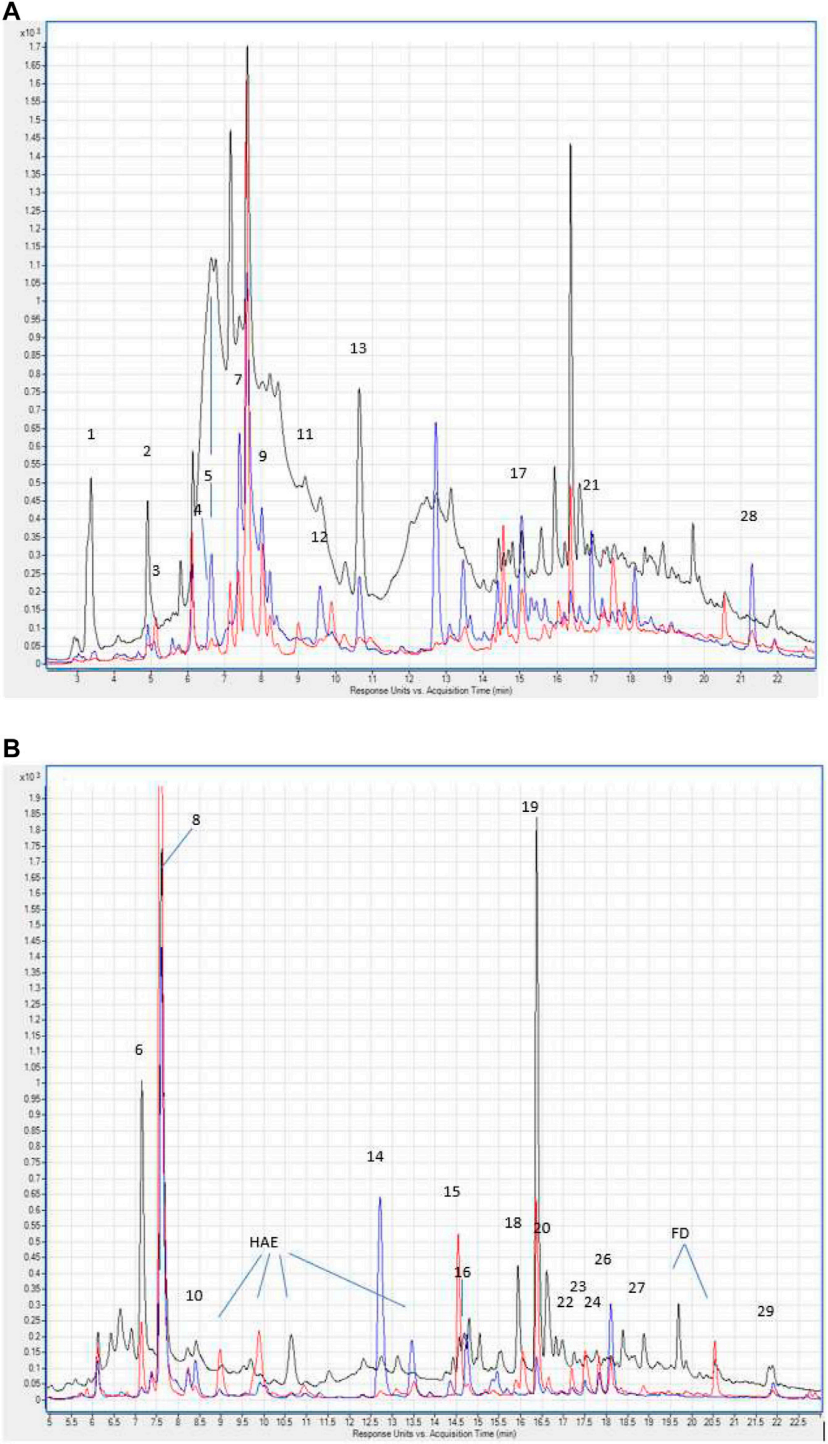
**(A)** Hydroxybenzoic acids and flavan-3-ols in the chromatograms of *Allium nutans* L. honey (red line), Helianthus annuus L. honey (blue line) extracts. λ 280 nm. Numbering corresponds to Table 1. Hydroxycinnamic acids and flavonols in the chromatograms of extracts Allium nutans L. (red line), Helianthus annuus L. (blue line). λ 325 nm. The numbering corresponds to Table 1. HAE - hydroxycinnamic acid esters. FD - flavonol derivatives.

The total content of phenolic compounds in the samples of the Allium nutans L. honey were compared to that of the Helianthus annuus L. honey, which was collected in the East Kazakhstan region during the same year. The results show that polyphenols extracted from Allium nutans L. honey, have significantly higher amount of quercetin, *p*-Coumaric acid, apigenin, kaempferol, pinobanksin, syringic acid, 4-Hydroxybenzoic acid than the extracts from the Helianthus annuus L. honey (Table 1) with the corresponding HPLC analyses shown in Figure 1A,B.

**TABLE 1:** Phenolic compounds in ethyl acetate extract of the honey *Allium nutans* L. and Helianthus annuus L. honey (mg/kg).

№	Phenolic compounds	*Allium nutans* L. Honey	Helianthus annuus L. honey
1	Gallic acid		
2	Protocatechuic acid	10 ± 2	26 ± 4.8
3	Tyrosol	6.4 ± 1.6	
4	4-Hydroxybenzoic acid		40 ± 3.8
5	Procyanidin B 1	26 ± 4	195 ± 5.9
6	3-*p*-Coumaroylquinic acid^*^	30 ± 4.3	
7	(+)-Catechin	113 ± 6.7	438 ± 5.6
8	Chlorogenic acid (5-Caffeoylguinic acid)	533 ± 11.2	240 ± 3.1
9	Procyanidin	179 ± 10.1	243 ± 11.2
10	*trans*-Caffeic acid	4.3 ± 0.8	13 ± 1.0
11	Syringic acid	Undetected	Undetected
12	Procyanidin B 2	13 ± 2.6	233 ± 6.5
13	(−)-Epicatechin	20 ± 1.8	222 ± 9.1
14	*trans*-*p*-Coumaric acid	3.7 ± 0.9	119 ± 3.5
15	Myricetin-glycoside	168 ± 8.9	
16	trans-Ferulic acid	3.9 ± 1.2	24 ± 3.3
17	Procyanidin	152 ± 12.6	327 ± 9.9
18	Quercetin-3-glucuronide	Undetected	Undetected
19	Quercetin-3-galactoside	186 ± 13.1	36 ± 3.9
20	Quercetin 3-glucoside	15 ± 3.2	6.2 ± 1.1
21	Procyanidin	Undetected	178 ± 7.8
22	Quercetin 3-xyloside	24 ± 2.2	7.9 ± 1.2
23	Quercetin 3-arabinopyranoside	55 ± 5.1	16 ± 2.2
24	Quercetin 3-arabinofuranoside	32 ± 2.2	20 ± 1.7
25	Kaempferol 3-glucoside	Undetected	Undetected
26	Quercetin-3-rhamnoside (quercitrin)	25 ± 3.3	83 ± 5.2
27	Myricetin	9.2 ± 1.2	Undetected
28	Procyanidin	32 ± 2.8	203 ± 6.4
29	Quercetin	9.4 ± 3.0	25 ± 2.2
30	Kaempferol	Undetected	0.7 ± 0.1
	Hydroxybenzoic acids	17 ± 2.2	65 ± 4.5
	Hydroxycinnamic acids	575 ± 6.9	397 ± 12
	Flavonols	524 ± 6.5	195 ± 5.5
	Flavan-3-ols	535 ± 8.3	2039 ± 16.5
	Total	1,651	2,696

The results are average of three independent experiments.

### Free Radical Scavenging Activity of Different Honey Extracts

Free radical scavenging activity of extracts from *Allium nutans* L. and Helianthus annuus L. honey were measured *in vitro* using two different biochemical assays, DPPH (Table 2) and ABTS (Table 3). While no difference was observed between the two extracts in the DPPH biochemical assay, the results from the ABTS assay showed that Allium nutans L*.* honey had twice as much scavenging activity as sunflower Helianthus annuus L. extracts (Table 3).

**TABLE 2:** The values of the optical density of DPPH radical solution.

Sample	Optical density	^a^Inhibition of the DPPH radical, %
*Allium nutans* L. honey	0.215 ± 0.05	78
Helianthus annuus L. honey	0.302 ± 0.09	70
Control (DPPH solution without test sample)	1,511	0

a The results are average of three independent experiments.

**TABLE 3:** Inhibition of ABTS + radical by honey extract in trolox equivalent (TE).

Sample	(TE)/ml
*Allium nutans* L. honey	91.6 ± 4.9*
Helianthus annuus L. honey	48.7 ± 6.1

*p* < 0.05 relative to control. The results are average of six independent experiments.

### **Cytoprotective Effect of** *Allium nutans L.* **Extracts**

The MTT results showed no cytotoxicity and the presence of pronounced cytoprotective properties in relation to the viability of isolated alveolar macrophages in the presence of the cytotoxic substance doxorubicin. An extract dilution of 1:10, significantly increased alveolar macrophages viability by 86% compared to the control (Table 4). The effect remained significantly high (28%) even at a higher dilution factor (in 1:100 dilution). Interestingly, 10% of honey extract showed a twofold increase in human fibroblast cell viability compared to that of the negative control (Table 5).

**TABLE 4:** Viability of rabbit alveolar macrophages in the MTT assay when using extract of the *Allium nutans* L. honey (*n* = 6).

Study groups	The relative values of viability as % of control	
	A dilution of 1:100	A dilution of 1:10
Control	100%	
The extract of the *Allium nutans* L. honey	128.2 ± 7.4	186.8 ± 9.9^a^
Doxorubicin 1 mcg/ml	52.4 ± 7.8^a^	
Doxorubicin 10 mcg/ml	18.4 ± 1.2^a^	
Doxorubicin 1 mcg/kg + extract of the *Allium nutans* L. honey	91.7 ± 6.6^b^	105.7 ± 9.4^b^
Doxorubicin 10 mcg/ml + extract of the *Allium nutans* L. honey	64.3 ± 4.1^b^	90.3 ± 8.6^b^

a Note *p* < 0.05 relative to control.

b *p* < 0.05 relative to doxorubicin.

**TABLE 5:** Viability of hFF in MTT assay when using the extract of the *Allium nutans* L. honey (*n* = 5).

Group name	Concentration (%)	The relative values of viability as % of control
Control	—	100
The extract of the *Allium nutans* L. honey	A dilution of 1:10	195 ± 10.6^a^

a Note: *p* < 0.05 relative to the control.

### Hepatic Injury and Cytoprotective Effects of Honey Extracts

The results on the effects of the studied substance, the Allium nutans L. honey extract, on biochemical parameters including the activity of aminotransferases, bilirubin, protein, and glucose levels in rats with acute CCl-induced hepatitis are shown in Table 6. In comparison to group 2 (untreated hepatic model), the experimental group showed significant improvements in the activity of both ALT and AST. However, the reminder of the tested biochemical parameters remained unchanged.

**TABLE 6:** The effect of the *Allium nutans* L. honey extract on biochemical parameters. hepatitis (*n* = 6).

Studied parameters	Group 1—negative control animals, *n* = 6	Group 2 - animals with CCl4-induced hepatitis, without treatment, (control), *n* = 6	Group 3-animals with CCl4-induced hepatitis that received the *Allium nutans* L. Honey extract, (test), *n* = 6
ALT, mmol/s*L	0.193 ± 0.044^a^	0.393 ± 0.045	0.296 ± 0.57^a^
AST, mmol/s*L	0.204 ± 0.009^a^	0.256 ± 0.024	0.214 ± 0.29^a^
Bilirubin mg/dL	0.50^a^ ± 0.099	0.579 ± 0.070	0.660 ± 0.135
Total protein (g/l)	57.4 ± 2.5	59.1 ± 1.4	63.1 ± 2.4
Glucose (mmol/l)	7.65 ± 0.14	7.37 ± 0.17	6.80 ± 0.16

a Notes: P<0.05 in relation to the control group.

The results are average of six independent experiments.

### Histological Analyses of Liver Samples

**FIGURE 2:**
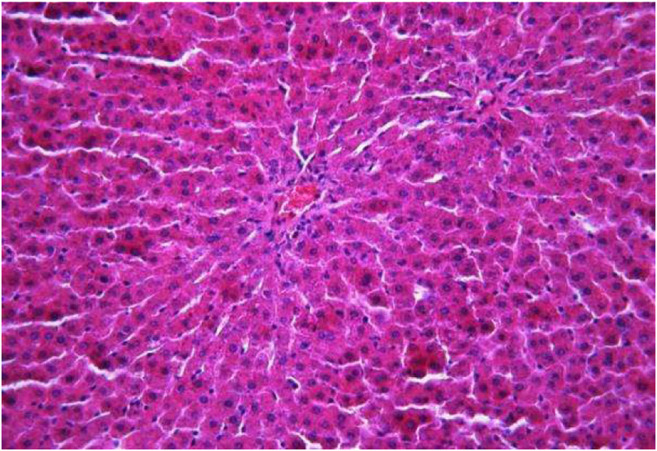
Liver tissue. Negative group. The structure of the liver lobule can be visualized well. Stain: hematoxylin and eosin. Magnification ×100.

**FIGURE 3:**
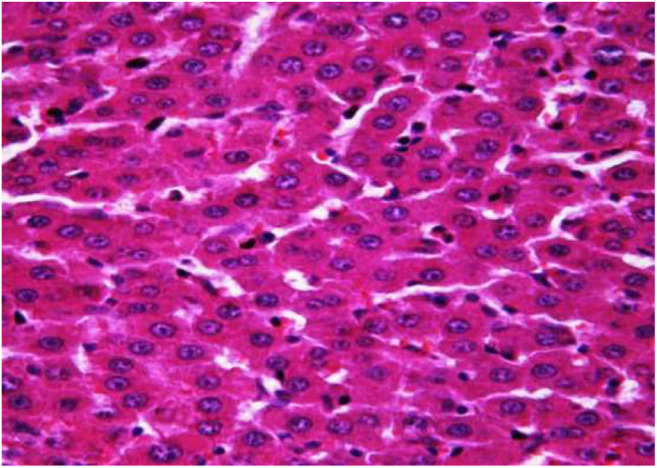
Liver tissue. Negative group. There is a linear cord structure of hepatocytes in the hepatic lobule. Stain: hemotoxin and eosin. Magnification ×400.

The morphological study of the liver tissue of negative control animals shows a well-visualized structure of hepatic lobules and a linear cord arrangement of hepatocytes in the hepatic lobule (Figures 2, 3). While, in the control group of the experiment, animals developed typical acute hepatitis (toxic hepatosis) seen from the significant changes in all structural components of the hepatic lobule and the disruption of blood circulation at the level of capillaries of liver sinusoids and periportal vessels.

**FIGURE 4:**
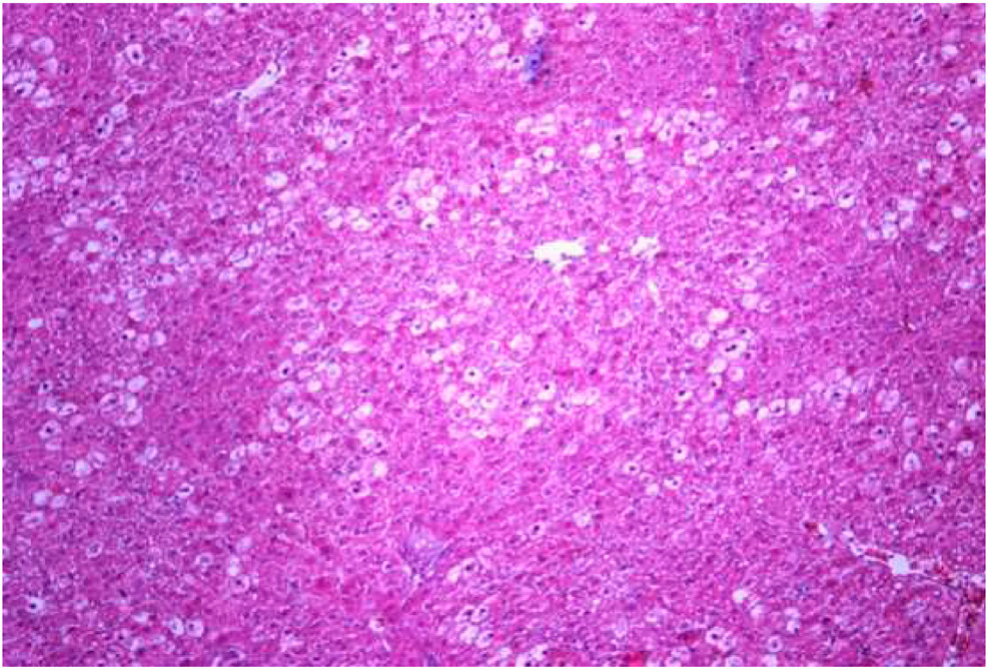
Liver tissue. Positive control group. The structure of hepatic lobule is broken; signs of hydropic and vacuolar dystrophy of hepatocytes. Stain: hemotoxin and eosin. Magnification ×100.

**FIGURE 5:**
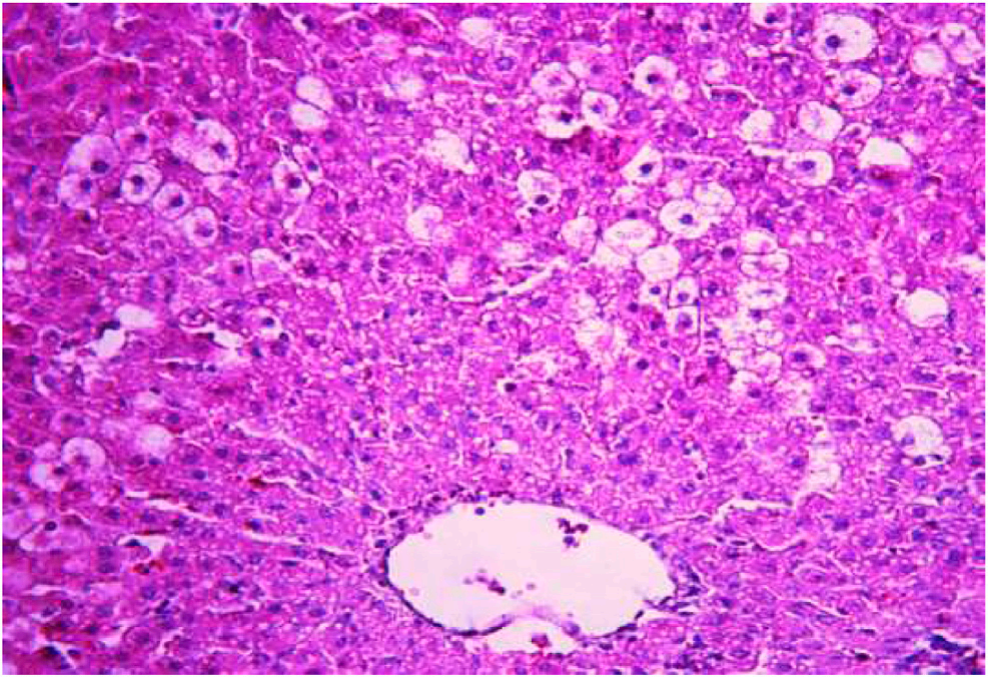
Liver tissue. Positive control group. Hydropic and vacuolar dystrophy of hepatocytes, mainly of the periportal zones of the hepatic lobule. Cytoplasmic vacuolization, pycnotic nuclei, focal necrosis of hepatocytes; the nuclei are lysed—“empty cells”. Stain: hemotoxin and eosin. Magnification ×200.

**FIGURE 6:**
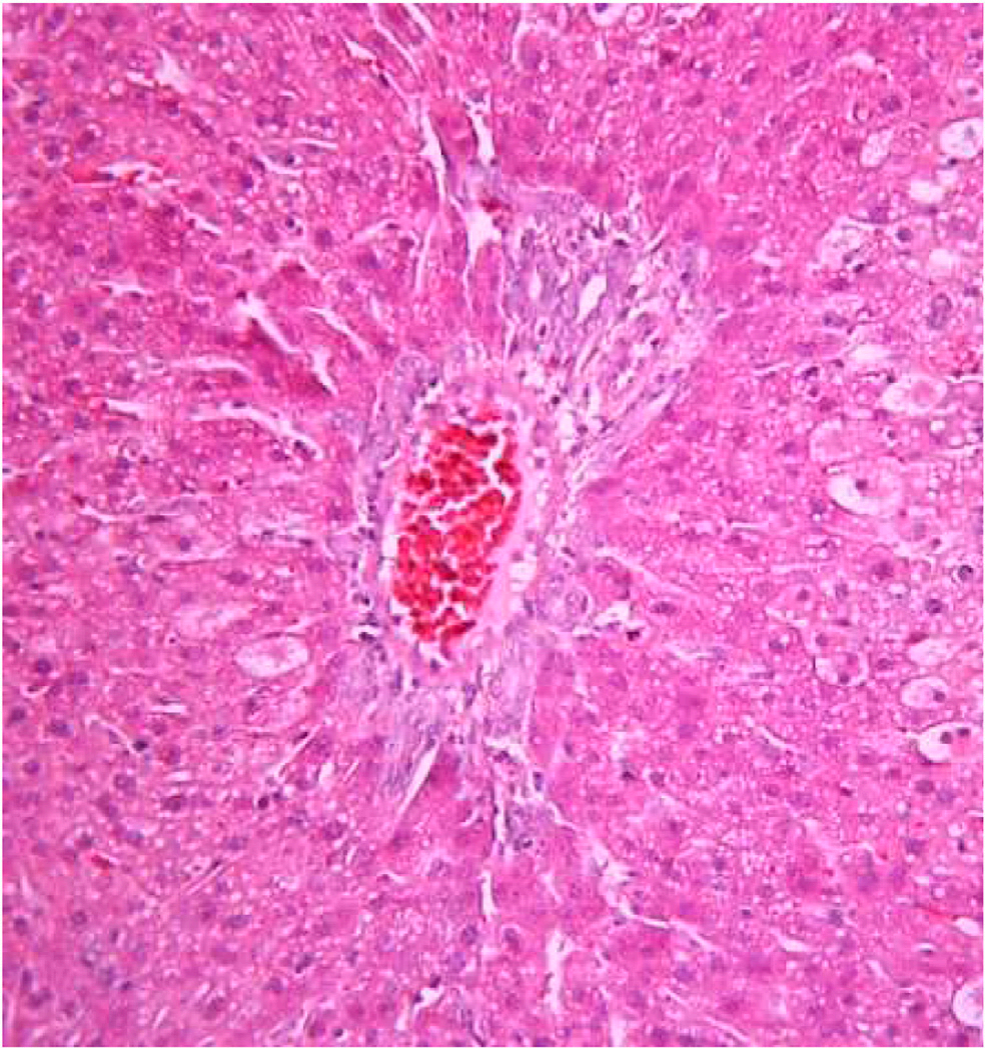
Liver tissue. Positive control group*.* Fullness of the periportal vessels. Hydropic and vacuolar dystrophy of hepatocytes of the periportal zones of the hepatic lobule. Stain: hematoxylin and eosin. Magnification ×200.

**FIGURE 7:**
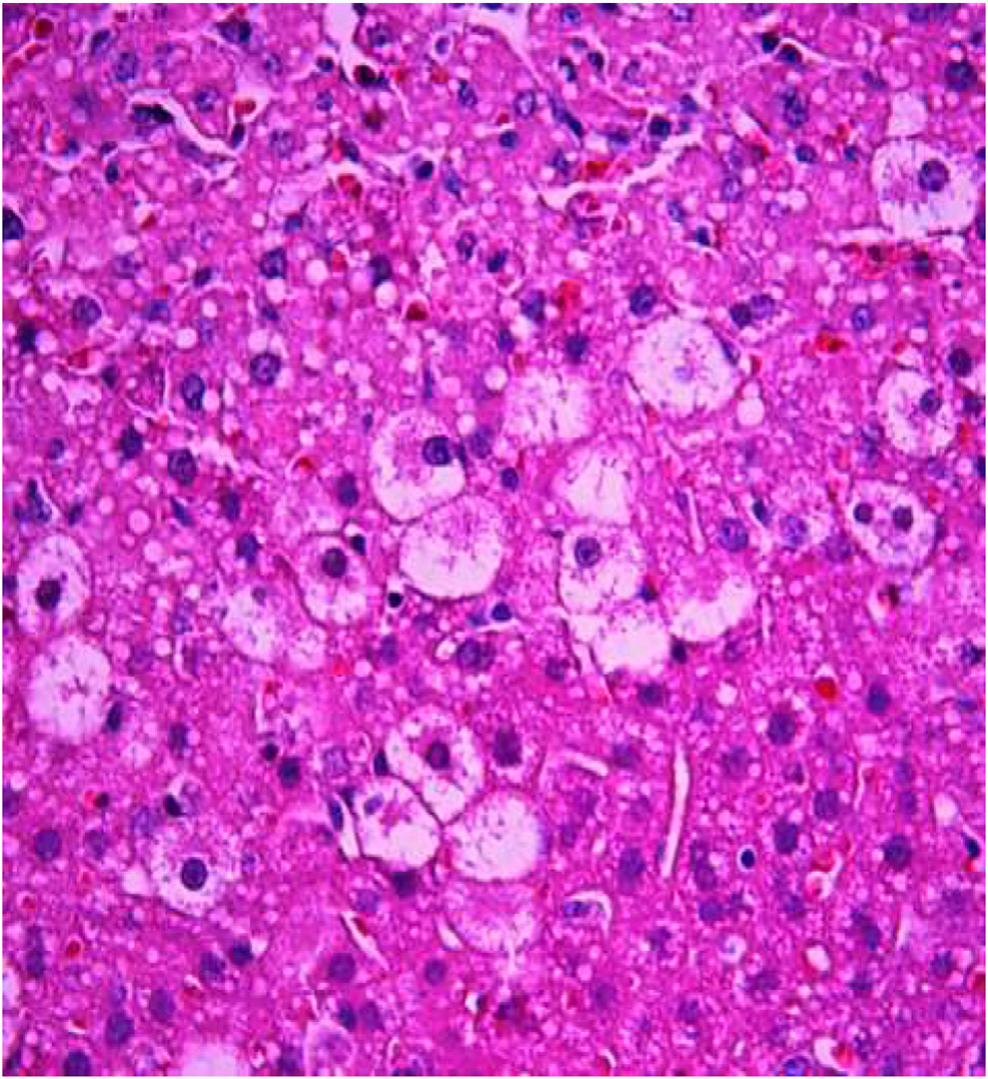
Liver tissue. Positive control group. Hyperemia of the capillaries in the sinusoids. Hydropic and vacuolar degeneration of hepatocytes. Stain: hematoxylin and eosin. Magnification ×400.

**FIGURE 8:**
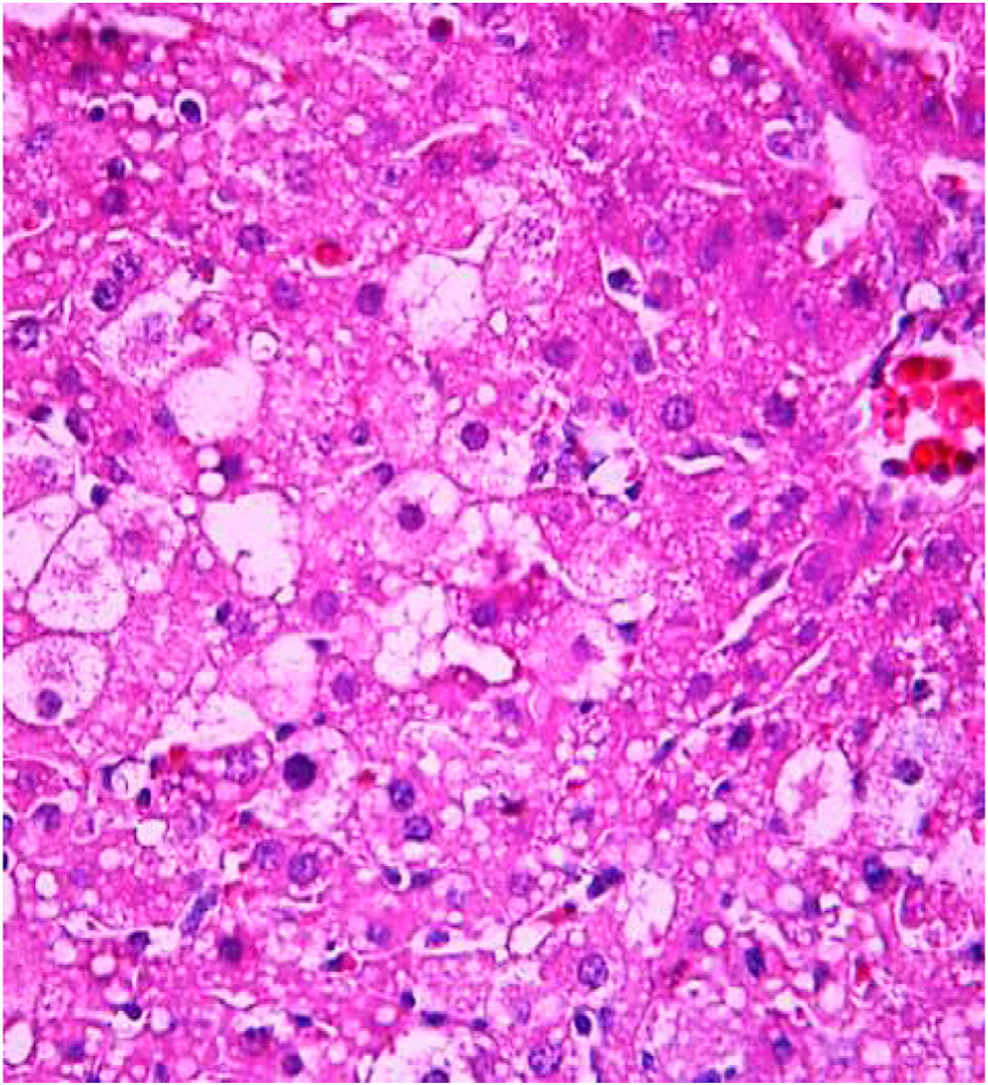
Liver tissue. Positive control group. Visible groups of hepatocytes in the state of intralobular focal necrosis, the cytoplasm contains light mesh-like formations. Stain: hemotoxin and eosin. Magnification ×400.

**FIGURE 9:**
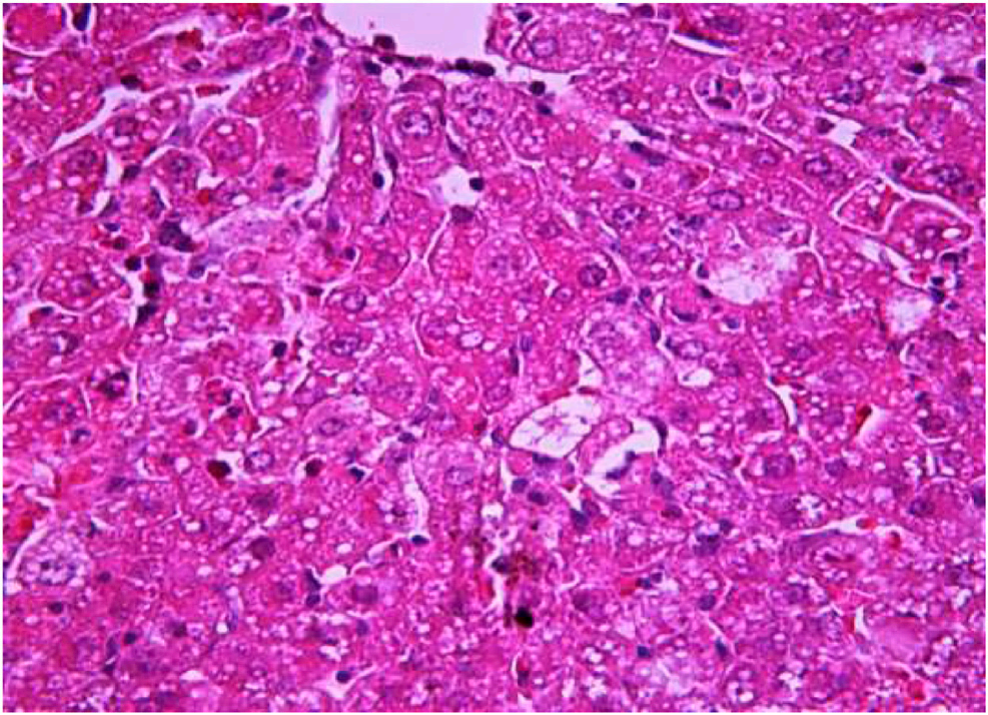
Liver tissue. The administration of the honey extract. The restoration of the linear cord structure of the liver lobule is noted. The central vein and capillaries of sinusoids are filled with blood. Stain: hemotoxin and eosin. Magnification ×100.

**FIGURE 10:**
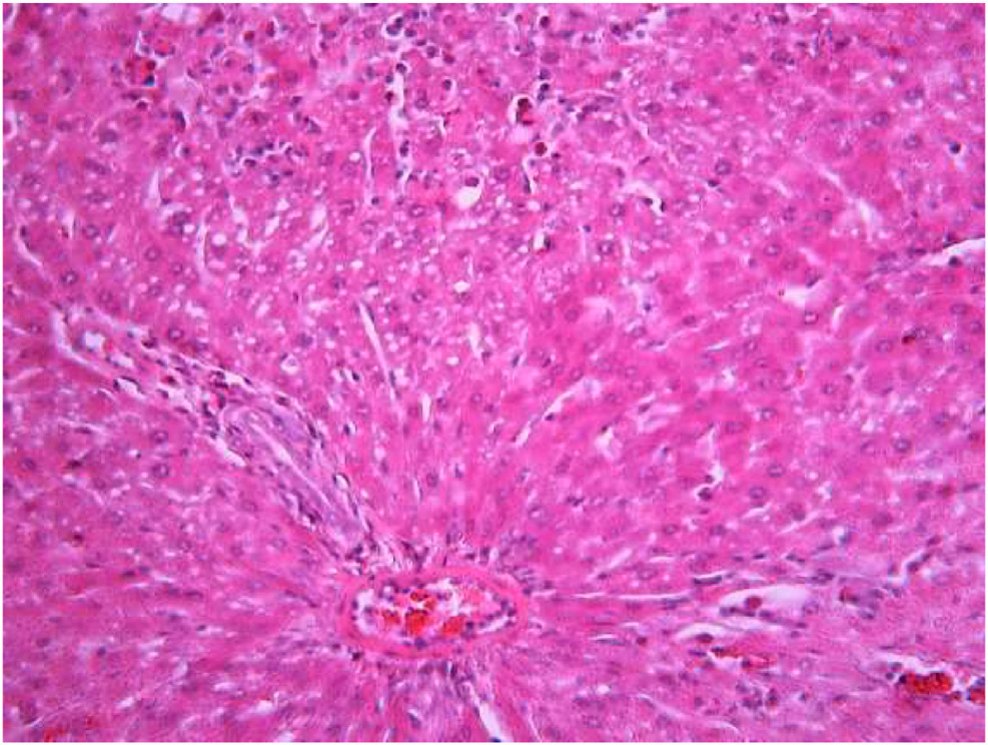
Liver tissue. The administration of the honey extract. Restoration of the linear cord structure of the hepatic lobule; the nuclei are located in the cell center; a few hepatocytes in the state of ballooning degeneration. Stain: hemotoxin and eosin. Magnification ×400.

The most typical structural changes were the development of hydropic and vacuolar dystrophy of hepatocytes in the periportal zones of the hepatic lobule. It shows enlarged groups of cells in the liver lobules with a well-visualized cell membrane; vacuolization of cytoplasm, which is heterogeneous and occasionally foamy; and pycnotic nuclei. In some vacuolized hepatocytes, the nuclei are driven to the periphery; foci of centralobular focal liquefactive necrosis are found; the nuclei are lysed and resemble “empty cells” (Figures 4−8). Furthermore, histopathological analyses of the Allium nutans L. honey treated group, showed significant reduction in the damages of structure of liver tissue. Specifically, the preservation of the linear cord structure of the hepatic lobule is noted; the nuclei in the cells are located mainly centrally; though there are a few hepatocytes in a state of vacuolar dystrophy, and the sinusoids are excessively filled with blood (Figures 9, 10).

## Discussion

Honey is a natural product of honeybees made up of a mixture of carbohydrates such as fructose, glucose, and sucrose. It also contains flavonoids acquired through contact with pollen, which are a rich source of secondary metabolites with high antioxidant activity (Tauber et al., 2019; Olas, 2020). It was shown to have potent therapeutic activities against several disorders including different types of cancers, diabetes, neurological and cardiovascular diseases (Afrin et al., 2018; Nguyen et al., 2019; Waheed et al., 2019; Olas, 2020). The therapeutic potential of honey is thought to be due its potent antioxidant activity. It is known that the antioxidant activity of honey is highly dependent on the characteristics of the region where honey is collected, this is well illustrated by the example of honey collected from different phytogeographic regions of Turkey (Doğan et al., 2014; Akyol et al., 2015). In the conditions of our experiment, we also revealed a significant superiority in the antiradical test DPPH and in the value of the trolox equivalent of honey Allium nutans L. (Siberian chives), which are perennial herbaceous plants endemic to the Altai Mountains region, compared to Helianthus annuus L. honey collected in southern region of Kazakhstan.

One of the objectives of the current work was to evaluate the protective effects of different honey types against CCl4-induced hepatotoxicity in rat models. The results showed that the Allium nutans L. honey has significant cytoprotective properties as demonstrated by the reduction of the degree of damage and protection of liver cells from CCl4-induced hepatitis, as well as the improvement of cell viability *in vitro* and the ability to protect isolated cells from the damage caused by cytotoxic compound, doxorubicin.

Furthermore, the increase in the serum concentration of ALT and AST in the untreated group compared to the negative control, suggests that a model of acute toxic hepatitis was obtained. Interestingly, the administration of the Allium nutans L*.* honey extract resulted in a significant reduction in the concentration of liver cytolytic enzymes-ALT and AST, as well as maintaining normal levels of bilirubin, total protein, and glucose, which were further confirmed by histological analyses.

It is well established that honey has significant antioxidant effects based on their polyphenolic composition (Pauliuc et al., 2020). The polyphenolic compounds shown in the composition of the Allium nutans L Allium nutans L. honey appear to contain a higher concentration than the most common Helianthus annuus L. honey. This finding is similar to that of Gośliński et al., which showed that (2020) (Gośliński et al., 2020) the higher antioxidant properties of Manuka honey (originating from New Zealand), compared to that of the Polish flower honey, are most likely to be due to the increased content of polyphenols.

Thus, the various polyphenols found in Allium nutans L. honey are the most likely reason of the relatively high antiradical activity of the Allium nutans L. honey, which we assumed to be due to their ability to bind free radicals as demonstrated by the DPPH and ABTS chemical assays. Consequently, the antiradical effect of honey against CCl4-generated free radicals is forms the basis for reducing the hepatotoxic effect observed in CCl4-induced heaptoxicity. Interestingly, the hepatoprotective property of the Allium nutans L. honey was evidently shown via reducing the cytolytic effect of CCl4 and preserving the morphological structure of hepatocytes. This property of the extract of honey Allium nutans L. is not limited to liver cells, rather appears to protect other cells, as confirmed by the *in vitro* studies of isolated rabbit macrophage cells and human fibroblasts.

CCl4-induced hepatitis is a widely used model for evaluating the hepatoprotective activity of drugs and various substances. The hepatotoxic effect of CCl4 results from the activity of cytochrome P450 2E1, causing the formation of a highly reactive trichloromethyl radical (CCl_3_), which then, in the presence of oxygen, turns into a more destructive trichloromethyl peroxide radical (CCl_3_OO) (Weber et al., 2003). In the present study, a single injection of CCl_4_, was able to induce a reliable model of acute hepatitis, which was confirmed by blood biochemical tests and liver histomorphology. The biochemical analyses showed a two-fold increase in the ALT activity compared to control, confirming the damage to hepatocyte membranes, an increased permeability, and cell death accompanied by a release of intracellular substances into the blood and lymph. The increase in the ALT activity, which is a cytoplasmic enzyme, compared to the activity of AST, which has a mitochondrial-cytoplasmic location, supports the fact that the damage occurred to the external membrane of the hepatocytes (Mantawy et al., 2012) (Zeliha, 2017).

The preliminary (7 days before the introduction of carbon tetrachloride) and continued (14 days after the introduction of the hepatotropic compound) administration of the extract of the Allium nutans L*.* honey to rats significantly improves the picture of hepatitis.

However, administration of the Allium nutans L*.* honey extract resulted in a significant decrease in the level of activity of ALT and AST enzymes The de Ritis coefficient value of the ratio of AST/ALT activity was 1.05 for negative control group, 0.65 for the positive control group, and 0.72 for the experimental group that received the honey extract. It is well known that the lower this coefficient, the more unfavourable is the prognosis for the course of the pathological process (Weber et al., 2003).

The extracts of both types of honey inhibit free radicals *in vitro* with a significantly higher anti-radical activity of Allium nutans L. compared to the Helianthus annuus L. extracts. The relatively high antioxidant and antiradical activity of the *Allium nutans* L. honey was further confirmed to have potential protective effect on the model of acute toxic hepatitis in rats (experimental group).

In conclusion, liver diseases are among the most serious disorders and prevention and treatment options are still limited, despite the huge progress in modern medicine. Although liver tissues are unique in their ability to regenerate, there is a probability of transition from inflammatory conditions to fibrosis or cirrhosis. The role of oxidative stress and inflammation in the pathogenesis of some liver diseases, such as hepatitis and hepatosis, is well-documented (Weber et al., 2003). Thus, inhibiting or slowing the chain reaction of oxidation and inflammation can be a promising therapeutic strategy for the treatment of liver damage.

Thus, our results in an *in vitro* study and on rats as a whole confirm the protective properties of honey both in relation to isolated cells and in relation to the liver of rats during intoxication. The results of our research draw attention to the protective properties of a unique and rare monofloral honey from the Altai Mountains region - Siberian chives honey.

Of course, it is necessary to conduct clinical studies to confirm the efficacy and safety of Allium nutans L. (Siberian chives) for the treatment of intoxication in humans.

## Data Availability

The raw data supporting the conclusion of this article will be made available by the authors, without undue reservation.
